# A system-based analysis of the genetic determinism of udder conformation and health phenotypes across three French dairy cattle breeds

**DOI:** 10.1371/journal.pone.0199931

**Published:** 2018-07-02

**Authors:** Andrew Marete, Mogens Sandø Lund, Didier Boichard, Yuliaxis Ramayo-Caldas

**Affiliations:** 1 Génétique Animale et Biologie Intégrative, INRA, AgroParisTech, Université Paris-Saclay, Jouy-en-Josas, France; 2 Center for Quantitative Genetics and Genomics, Aarhus University, Tjele, Denmark; University of Illinois, UNITED STATES

## Abstract

Using GWAS to identify candidate genes associated with cattle morphology traits at a functional level is challenging. The main difficulty of identifying candidate genes and gene interactions associated with such complex traits is the long-range linkage disequilibrium (LD) phenomenon reported widely in dairy cattle. Systems biology approaches, such as combining the Association Weight Matrix (AWM) with a Partial Correlation in an Information Theory (PCIT) algorithm, can assist in overcoming this LD. Used in a multi-breed and multi-phenotype context, the AWM-PCIT could aid in identifying udder traits candidate genes and gene networks with regulatory and functional significance. This study aims to use the AWM-PCIT algorithm as a post-GWAS analysis tool with the goal of identifying candidate genes underlying udder morphology. We used data from 78,440 dairy cows from three breeds and with own phenotypes for five udder morphology traits, five production traits, somatic cell score and clinical mastitis. Cows were genotyped with medium (50k) or low-density (7 to 10k) chips and imputed to 50k. We performed a within breed and trait GWAS. The GWAS showed 9,830 significant SNP across the genome (p < 0.05). Five thousand and ten SNP did not map a gene, and 4,820 SNP were within 10-kb of a gene. After accounting for 1SNP:1gene, 3,651 SNP were within 10-kb of a gene (set1), and 2,673 significant SNP were further than 10-kb of a gene (set2). The two SNP sets formed 6,324 SNP matrix, which was fitted in an AWM-PCIT considering udder depth/ development as the key trait resulting in 1,013 genes associated with udder morphology, mastitis and production phenotypes. The AWM-PCIT detected ten potential candidate genes for udder related traits: *ESR1*, *FGF2*, *FGFR2*, *GLI2*, *IQGAP3*, *PGR*, *PRLR*, *RREB1*, *BTRC*, and *TGFBR2*.

## Introduction

Genetic architecture of complex phenotypes in cattle includes many loci affecting a given trait [1]. Most of these loci have small effects, but few segregating loci have moderate-to-large effects possibly due to epistatic effects, varying selection goals or recent selection for the favorable mutant allele. Moreover, markers collectively capture most but not all additive genetic variance for phenotypes. The incomplete variance capture may be due to causal mutations with low allele frequencies and therefore in incomplete linkage disequilibrium (LD) with markers [2]. To reduce this LD, we can either do a between breed analysis with a large sample of genotyped cows [3] or combine the results of a within breed GWAS in a multi-breed context. This is possible because the cost of genotyping is decreasing thus allowing many breeds, including those of medium population size, to be genotyped rapidly primarily for genomic selection purpose. These large populations of genotyped cows with own performances allows us to: (1) detect QTL for newly recorded traits or traits previously not studied; (2) carry out large confirmation studies for conventional traits. Previous studies have demonstrated that polymorphic sites that segregate within and across bovine populations can be studied using imputed low-to-dense genotypes [4,5]. Such genotypes have been used in model organisms and dairy cattle leading to the identification of candidate causal variants or closely neighboring variants that control complex phenotypes [6,7]. These studies have been useful for identifying QTL regions and probable genes associated with a phenotype. So far, however, there have been few validation studies of the vast number of putative variants across and between breeds and amongst multiple phenotypes. This study uses 50k SNP data to validate such variants using the Association Weight Matrix (AWM) [8] approach as a post GWAS analysis tool. The AWM is a systems biology approach for the genetic dissection of complex traits based on applying gene network theory to the results from GWAS. Hence, if the AWM SNP matrix is used in combination with a Partial Correlation (PC) in an Information Theory (IT) framework, and for correlated phenotypes, then it is possible to generate gene networks with regulatory and functional significance for udder related phenotypes.

Despite the limitations of the chip density, previous studies have shown the usefulness of the AWM to identify candidate genes in cattle, e.g. [9–10] and corroborated across different species, e.g. [11–12] in independent studies using the 50k marker density.

In this study, we report results based on GWAS analysis for mammary conformation, milk production and health phenotypes for 78,440 dairy cows. A multi-step validation by combining the results of single SNP, single phenotype, in a multiple-breed context using the AWM-PCIT algorithm was performed. The aim was to identify the genes associated with mammary conformation and health phenotypes in Holstein, Montbeliarde, and Normande breeds, accounting for milk production as supportive traits. We further explored gene networks with the main gene ontology domains including biological processes, cellular component, and molecular functions.

## Material and methods

### Phenotypes

The cow sample was comprised of 46,732 Holstein, 20,141 Montbeliarde, and 11,965 Normande all with known parents. Phenotypes were yield deviations as produced by French national evaluation system [13]. A yield deviation is a performance adjusted for all non-genetic effects of the model [14]. In case of repeated records, a yield deviation is adjusted for the permanent environmental effect and averaged per animal. The 12 traits were: fore udder attachment (FUA), udder depth or development (UDD), udder cleft (UC), udder balance (UB), front teat placement (FTP), milk (MY), fat (FAT and FAT%), protein (PROT and PROT%) clinical mastitis (CM) and somatic cell score (SCS). SCS are derived from Somatic Cell Counts (SCC). SCC are obtained at each monthly test-day, and SCS are defined as SCS = 3+log_2_(SCC/100,000). CM events are declared by the farmer and recorded by the technician at each test-day. The analysis trait is defined by 1 if the cow has at least one clinical mastitis event in the lactation, and 0 if no there is no event. SCS and CM are repeated over lactations. The model for obtaining yield deviations was different according to the trait and all models included the genetic additive effect. The model for SCS and CM (recorded in the first three lactations), included the effect of herd x year, age at calving x parity x year, month of calving x parity x year, preceding days dry (for later parities), and a permanent environmental effect. All models assumed heterogeneous variances depending on herd-year. Each cow had one record for each trait. UDD can be either udder depth (Holstein and Normande) or udder development (Montbeliarde), but treated as the same trait because they have similar definitions. Depending on the trait, the number of animals with phenotypes ranged from 7,671 to 11,965 in Normande, 13,879 to 20,141 in Montbeliarde, and 32,491 to 46,732 in Holstein (Table 1). We estimated variance components by fitting a multiple trait REML animal model as implemented in the DMU software [15]. This study is based on already existing data hence we did not require ethical approval.

**Table 1:** Characteristics of udder conformation, production and health traits in three French dairy breeds.

Trait	Cows with traits			Standard Deviation		
	MON^1^	NOR^2^	HOL^3^	MON^1^	NOR^2^	HOL^3^
Fore Udder Attachment (FUA)	17,330	7,671	32,491	1.18	1.26	1.19
Udder Depth or Development (UDD)	17,330	7,671	32,491	0.98	0.81	0.84
Udder Cleft (UC)	17,330	7,671	32,491	1.26	1.28	0.85
Udder Balance (UB)	17,330	7,671	32,491	0.79	0.82	1.39
Front Teat Placement (FTP)	17,330	7,671	32,491	1.11	1.14	0.96
Milk Yield (MY)	20,096	11,944	46,732	786	742	991
Fat Yield (FAT)	20,096	11,944	46,732	33.7	34.7	40.2
Protein Yield (PROT)	20,096	11,944	46,732	27.5	25.7	30.1
Fat Percent (FAT %)	20,096	11,944	46,732	1.26	1.28	0.85
Protein Percent (PROT %)	20,096	11,944	46,732	1.28	1.28	0.85
Clinical Mastitis (CM)	13,879	9,013	32,491	0.24	0.26	0.36
Somatic Cell Score (SCS)	20,141	11,965	46,732	0.93	0.90	0.96

^1^Montbeliarde;

^2^Normande;

^3^Holstein

### Genotyping, quality control, and imputation

All cows were genotyped using Illumina BovineSNP50 BeadChip (50k) or Illumina BovineLD v.2 BeadChip. We used the UMD3.1 assembly of the bovine genome [16] for SNP chromosomal positions. We did not consider mitochondrial, X-chromosomal and Y-chromosomal SNP, as well as unmapped SNP for further analyses. We examined 43,800 SNP currently used in French genomic evaluation procedure [13]. Selection criteria was: call rate higher than 99%, minor allele frequency (MAF) higher than 2% in at least one of the three breeds, lack of Hardy–Weinberg Equilibrium (P < 10^−4^), technical quality assessed by their very low rate of Mendelian mismatch between parents and progeny and known position of genome assembly. We imputed cows genotyped from BovineLD v.2 BeadChip to 50k to obtain a genotype without missing information. This step was performed within breed in the conventional pipeline for genomic selection [13]. We imputed using FImpute software [17], and reference population included all 50k genotyped male and female animals per breed. Imputation error rate, measured in routine evaluation situation (and not in this study), varied from 0.2 to 2.5% depending on whether parents were genotyped. Across breeds, best imputation accuracies were observed in Montbeliarde which has the highest proportion of sires and dams genotyped with 50k, while accuracies were lower in Holstein, a breed with a smaller portion of genotyped dams, a lower percentage of 50k genotyped parents, and even a non-zero proportion of non-genotyped (usually foreign) sires.

### Statistical framework

GWAS was done within breed with the Mixed Linear Model Association (MLMA) method as implemented in GCTA [18]. Because phenotypes were yield deviations already adjusted for non-genetic effects, we did not consider additional fixed effects. For each phenotype and SNP i, the model used in each breed was the following

$$y\;=\;1\;\mu +Z\;u+w_{i}\;s_{i}+e$$ (1)

where **y** is a vector of yield deviations, μ is a mean; **u** is a vector of random additive polygenic effects and is $\sim N(0,\;G{\;\sigma }_{u}^{2})$ where **G** is genomic relationship matrix based on all cows with phenotypes per breed and all autosomes. **Z** is incidence matrix relating phenotypes **y** to **u**, **w**_**i**_ is a vector of genotypes for SNP i, s_i_ is the effect of SNP i, and **e** is a vector of random residual effects. We calculated the relationship between two individuals’ *j* and *k* as $\;g_{jk\;}\;=\;\frac{1}{W}\;\sum_{i\;=\;1}^{W}\frac{{(x}_{ij}\;-\;{2p}_{i})(x_{ik}-{2p}_{i})}{{2p}_{i}({1-\;p}_{i})}\;$, *x*_*ij*_ being the number of alleles for individual *j* and SNP *i* and *p*_*i*_ is the observed allelic frequency, and, *w* was 43,800. We applied a genome-wide Bonferroni correction on all 43,800 tests to account for multiple testing.

### Candidate variant discovery

We used the Association Weight Matrix (AWM) procedure to identify candidate genes per breed [8]. The AWM is a multiple trait approach that considers the genetic contribution of correlated traits allowing selection of pleiotropic SNP associated with numerous traits rather than a single trait. We classified trait information as either key or supportive trait, and the key trait in this study was udder depth or development (UDD) which is the most important type trait with the strongest relationship with mammary health and longevity. In addition, UDD is an aggregate trait, combining size, attachments, balance and strength of support. Populating the AWM starts with the selection of significant SNP from a GWAS [19]. The SNP additive effects are z-scored normalized by deviating the allele substitution effects from their mean and dividing by their standard deviation. We then created two matrices: (a) A z-scored additive values matrix (b) The GWAS p-values matrix. In both cases, rows represent SNP and columns represent traits. We then processed these matrices using the AWM algorithm, which includes five steps: (1) Primary SNP Selection: We select SNP associated with key trait using a P-value threshold (P < 0.05). (2): Exploring the dependency among traits: For the SNP selected in step (1), and, for the same threshold (P < 0.05), we register the average number of non-key traits to which the SNP are associated. In this study, that number was five traits. (3): Secondary SNP Selection: We select SNP from step (1) associated with at least five other traits including at least two udder traits. This step depends on correlation amongst traits and allows capturing most SNP associated with remaining traits. (4): Exploiting the genome map: We annotated the SNP captured in step (1), and step (3) using the UMD3.1 Genome assembly [16]. We classified the SNP that (i) mapped a gene, (ii) <10-kb to known genes, and, (iii) >10-kb to any coding region. For genes represented by more than one SNP, we select the SNP associated with the highest number of traits and has the lowest P-value average across all traits as representative for that gene. (5): Populating the AWM: Each {i,t} cell value in the AWM matrix corresponds to the z-score normalized additive effect of the i^th^ SNP on the t^th^ trait. This allows exploration of trait correlations column-wise, and gene/SNP interactions row-wise. We then calculate the SNP-based correlations and compare the SNP-based and genetic correlations, the latter being calculated as pedigree-based restricted maximum likelihood (REML), established in the same cows’ populations. We then use the AWM SNP matrix as the input for the PCIT algorithm [20] and for any trio of SNP; we estimate the first order partial correlation coefficients to identify meaningful gene-gene interactions. We annotated and clustered gene ontology (GO) annotations for significant PCIT gene-gene interactions using Cytoscape [21]. Finally, we compare the gene clusters amongst the three breeds and plot the most significant cluster.

## Results

### GWAS for all traits

**Fig 1:**
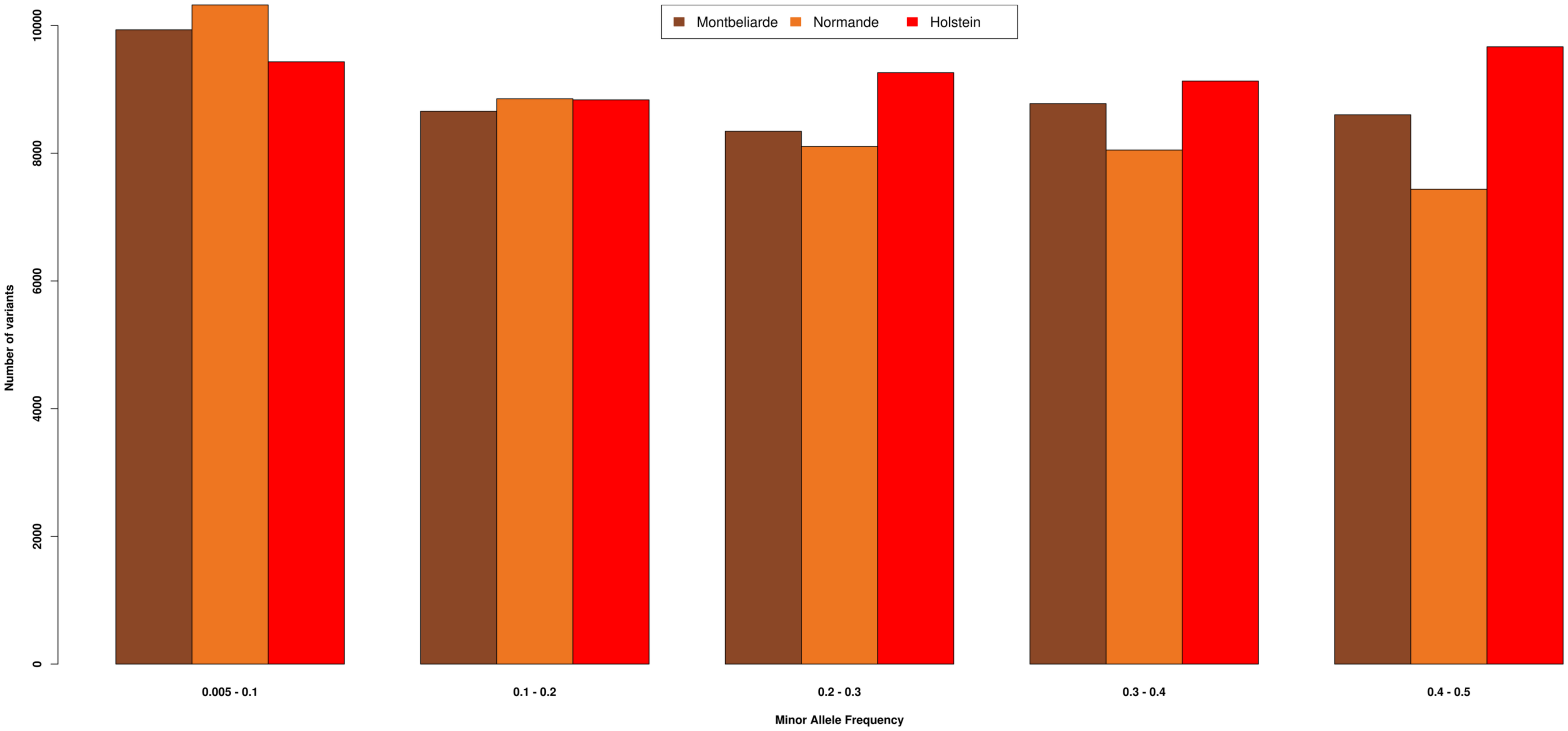
SNP variants as expressed in MAF in three French dairy breeds. The minor allele frequency of SNP variants in three French dairy breeds.

Collectively for 78,440 cows, imputation resulted in a genotype density of 43,800 SNP. Of these, 38,827, 38,109, and 40,810 SNP had a MAF greater than 0.1 in Montbeliarde, Normande, and Holstein. Distribution of allele frequencies (MAF) of imputed genotypes was almost uniformly distributed across the MAF classes (Fig 1).

**Fig 2:**
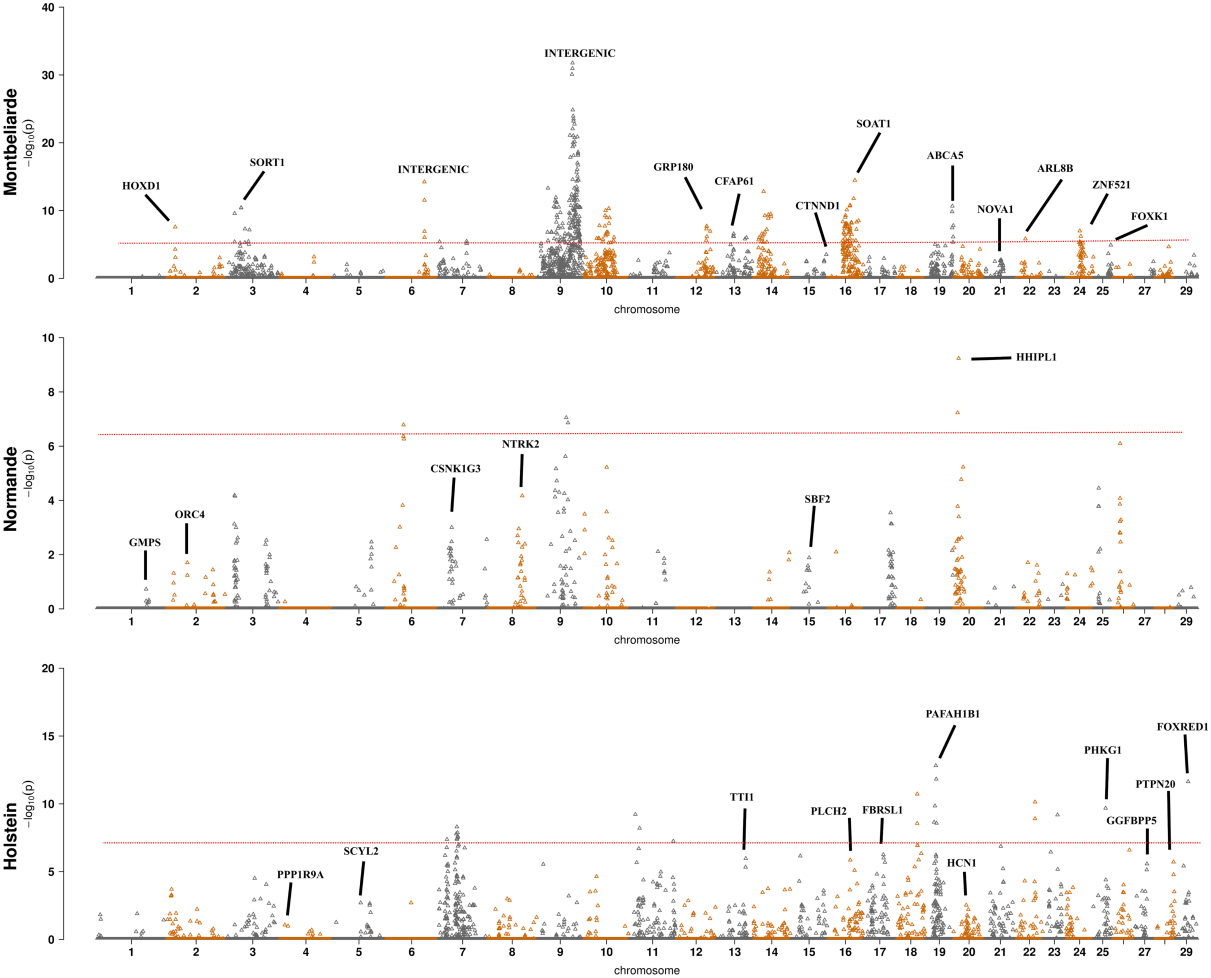
Manhattan plots for the key trait: Udder depth (Holstein and Normande) or development (Montbeliarde breed).

**Fig 3:**
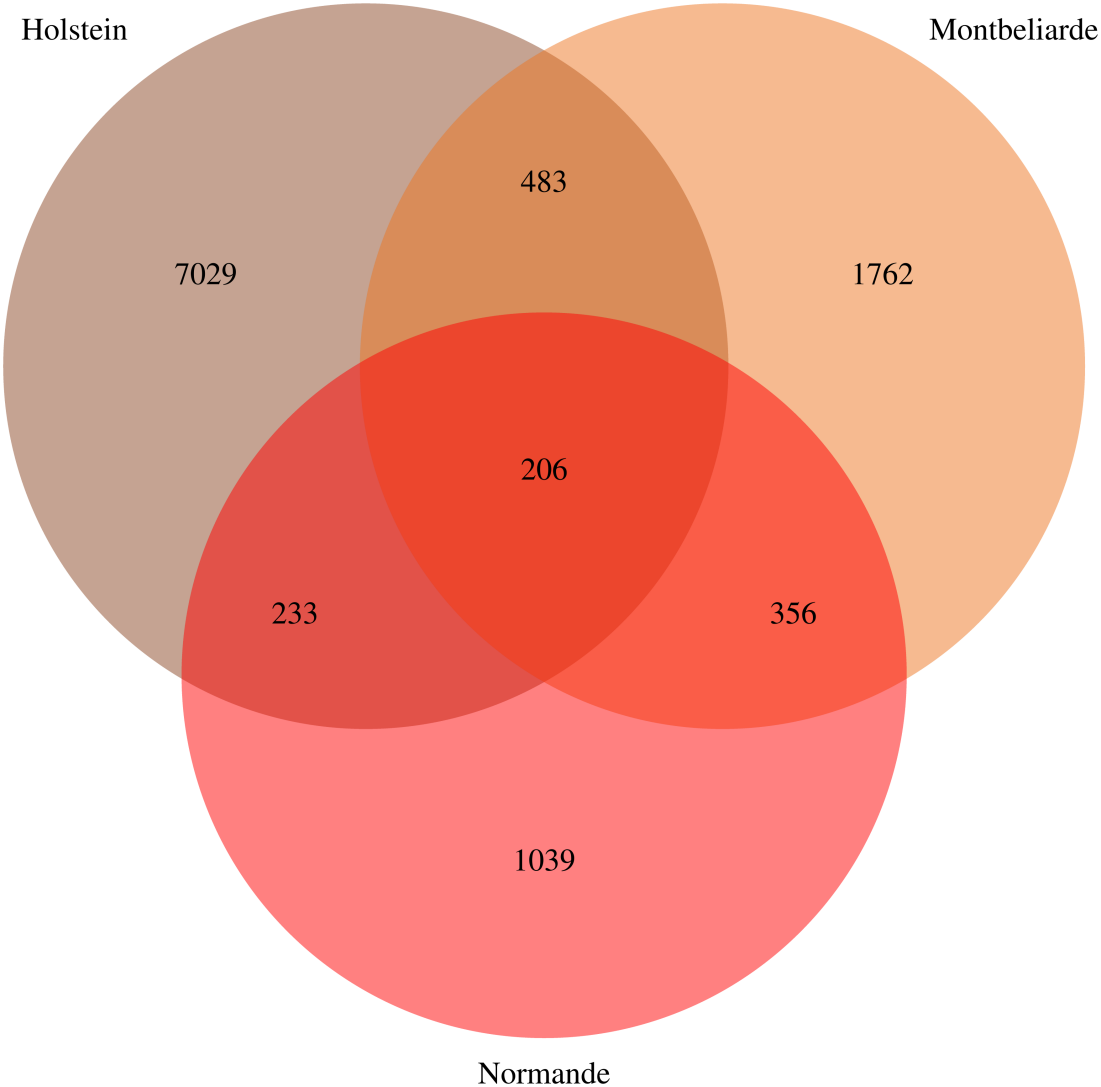
Summary of GWAS significant SNP and breed overlap for 12 traits in three French dairy breeds.

We performed GWAS for real and imputed SNP and yield deviations (YD) for 12 traits: five udder conformation traits, five milk production phenotypes, somatic cell score and clinical mastitis (Table 2). Fig 2 presents Manhattan plots for the key trait (Udder depth or development) for three breeds and S1 Fig for other traits. As expected, the number of significant SNP increased with breeds sample size. Holstein had 7,029 associated SNP, Montbeliarde had 1,762 associated SNP, and, Normande had 1,039 associated SNP (Table 2). There was an overlap of significant SNP across the breeds. Some 483 SNP were common between Holstein and Montbeliarde, 356 SNP were common between Holstein and Normande, 233 SNP were common between Montbeliarde and Normande, and 206 SNP were common among three breeds (Fig 3). We observed overlap of significant SNP between udder and milk production traits. Irrespective of the breed, 15 SNP overlapped in udder related traits, and 205 SNP overlapped in milk production phenotypes. When considering 1SNP:1Gene, 3,651 significant SNP were close to genes (<10-Kb) across three breeds. Of these, 1,017 were highly associated with udder conformation traits, 2,502 with production traits and 132 with somatic cell score and clinical mastitis. The 2,673 additional SNP satisfying step 4 of the AWM algorithm (as described in M&M) was augmented with the 3,651 significant SNP from GWAS forming the AWM matrix with 6,324 SNP (S1 Table). Of these, 1,309 SNP were common across three breeds, and they mapped 1013 genes for 12 traits.

**Table 2:** Summary of GWAS for udder related traits in three French dairy cattle breeds.

Trait^2^	Significant SNP^1^			Significant SNP close^3^ to gene		
	MON^4^	NOR^5^	HOL^6^	MON^4^	NOR^5^	HOL^6^
Fore Udder Attachment	45	29	558	32	20	235
Udder Depth or Development	204	38	57	84	13	47
Udder Cleft	37	24	402	29	22	237
Udder Balance	100	17	199	58	16	98
Front Teat Placement	125	37	45	64	29	33
Milk Yield	70	53	684	30	42	182
Fat Yield	68	47	515	31	41	112
Protein Yield	5	10	362	2	6	137
Fat Percent	481	390	1,498	136	105	480
Protein Percent	613	382	2,362	214	168	816
Clinical Mastitis	14	12	336	5	6	113
Somatic Cell Score			11			8

^1^Significant SNP = A SNP that has satisfied the Bonferroni threshold for a trait;

^2^Trait = A yield; deviation: phenotype corrected for environmental variances;

^3^Significant SNP within 10-kb of gene;

^4^Montbeliarde;

^5^Normande;

^6^Holstein

Significant SNP associated with the key trait were evident for Holstein and Montbeliarde, and the most significant SNP that mapped a gene for the key trait is presented per chromosome in Table 3. In total, 17 SNP were most significant per chromosome SNP and mapped to a gene in Holstein and Montbeliarde. This included eight for Montbeliarde, nine for Holstein. Two lead SNP in Montbeliarde were rs41640614 (BTA16, *SOAT1* gene, p = 3.47x10^-15^, Effect size = -0.185(0.02), MAF = 0.08) and rs108972236 (BTA19, *ABCA5* gene, p = 2.20x10^-11^, Effect size = -0.112(0.01), MAF = 0.20). The two lead SNP in Holstein were rs41641987 (BTA19, *PAFAH1B1* gene, p = 1.51x10^-13^, Effect size = -0.128(0.02), MAF = 0.04) and rs110651226 (BTA29, *FOXRED1* gene, p = 2.30x10^-12^, Effect size = 0.059(0.01), MAF = 0.45).

**Table 3:** Most significant SNP per chromosome associated with udder depth/development and mapping a gene.

Breed	SNP^1^	BTA^2^	Pos^3^ (bp)	Effect allele	MAF^4^	Effect size	SE Effect size	p^5^	Gene^6^
Montbeliarde	rs43293677	2	20760409	G	0.451	0.084	0.01	2.60X10^-08^	HOXD1
Montbeliarde	rs29019267	3	34184021	A	0.367	-0.092	0.01	3.67X10^-11^	SORT1
Montbeliarde	rs43704946	12	69648659	G	0.226	-0.095	0.01	1.97X10^-08^	GPR180
Montbeliarde	rs109080985	13	40031719	A	0.46	0.075	0.01	2.63X10^-07^	CFAP61
Montbeliarde	rs110761656	15	82317986	A	0.364	0.066	0.01	3.15X10^-04^	CTNND1
Montbeliarde	rs41640614	16	62100110	A	0.08	-0.185	0.02	3.47X10^-15^	SOAT1
Montbeliarde	rs108972236	19	61919633	C	0.201	0.112	0.01	2.20X10^-11^	ABCA5
Montbeliarde	rs41256881	22	21326038	G	0.315	0.078	0.01	1.49X10^-06^	ARL8B
Montbeliarde	rs42049077	24	31765644	T	0.105	0.127	0.02	1.00X10^-07^	ZNF521
Holstein	rs109049511	13	67557015	T	0.272	-0.051	0.01	1.07X10^-06^	TTI1
Holstein	rs41808096	16	51621826	C	0.199	0.059	0.01	1.39X10^-06^	PLCH2
Holstein	rs110859130	17	45680965	T	0.035	-0.128	0.02	5.57X10^-07^	FBRSL1
Holstein	rs41641987	19	24136906	A	0.13	-0.088	0.01	1.51X10^-13^	PAFAH1B1
Holstein	rs42067431	25	28003780	C	0.413	-0.055	0.01	2.08X10^-10^	PHKG1
Holstein	rs41565991	27	27804403	A	0.194	-0.059	0.01	2.66X10^-06^	GGFBPP5
Holstein	rs42147106	28	42881677	T	0.438	-0.047	0.01	1.94X10^-06^	PTPN20
Holstein	rs110651226	29	30003729	A	0.45	0.059	0.01	2.30X10^-12^	FOXRED1

^1^SNP = Reference SNP ID as assigned by NCBI;

^2^BTA = *Bos taurus* autosome;

^3^Position = base pair position in BTA (UMD3);

^4^MAF = minor allele frequency;

^5^p = Bonferroni corrected P-value;

^6^Gene = SNP annotation mapping a gene

We observed SNP associated with FUA and FTP in all breeds. The most significant of these signals were in Holstein and Montbeliarde and they include: BTA17 (rs41609100, F*GF2* gene, Effect size = -0.12(0.01), p = 4.95x10^-12^, MAF = 0.073, Holstein), BTA20 (rs109428015, *PRLR* gene, Effect size = -0.22(0.03), p = 3.16x10^-16^, MAF = 0.033, Holstein), and, BTA26 (rs42088948, *BTRC* gene, Effect size = -0.10(0.01), p = 6.89x10^-4^, MAF = 0.068, Montbeliarde).

### Genetic parameters and genomic AWM-based correlations

Heritability coefficients (diagonal), pedigree-based genetic correlations (upper diagonal), and SNP correlations (lower diagonal) are presented in Table 4. Heritability values are close to reported values for type traits [22], Fore udder attachment, (FUA) is 0.34, 0.26, 0.33 for Montbeliarde, Normande, and Holstein and higher for the other traits. For example, for Milk yield (MY), they reached 0.50, 0.61, and 0.54 in Montbeliarde, Normande and Holstein, respectively. These high values reflect the nature of the yield deviations (YD), which is a mean of records for repeated traits. YD is adjusted for permanent environment effect and thereby has a reduced non-genetic variability. As an example, assuming additive genetic variance of milk yield is 0.3, permanent environment variance is 0.2, and residual variance is 0.5 (we divide the residual variance by 2.5 records on average for production), the heritability of the corresponding YD is 0.3 / (0.3 + 0.5/2.5) = 0.6. These high values are very favorable for GWAS detection power.

**Table 4:** Heritability (diagonal), genetic^1^ (upper-diagonal), and SNP^2^ (lower-diagonal) correlations of udder conformation, milk production and health traits in three French dairy breeds.

Breed	Trait^3^	FUA	UDD	FTP	UB	UC	MY	FAT	PROT	FAT%	PROT%	CM	SCS
Montbeliarde	FUA	0.34	-0.11	-0.28	0.40	0.37	0.42	0.43	0.39	0.03	-0.05	0.19	-0.05
	UDD	0.54	0.37	-0.39	-0.38	0.37	0.33	0.32	0.31	-0.02	-0.08	0.09	0.03
	FTP	0.04	-0.01	0.43	-0.24	-0.15	0.20	0.22	0.21	0.06	0.04	0.06	0.02
	UB	0.59	0.66	-0.21	0.36	-0.03	0.02	0.01	0.00	0.00	-0.03	-0.09	-0.02
	UC	0.65	0.61	-0.05	0.4	0.30	-0.01	-0.01	-0.01	-0.05	-0.06	0.00	-0.05
	MY	0.33	0.1	-0.05	0.03	0.17	0.50	0.88	0.94	-0.22	-0.13	0.02	0.02
	FAT	-0.02	-0.07	0.08	-0.1	-0.06	0.31	0.44	0.90	0.26	0.09	-0.01	0.02
	PROT	-0.01	-0.09	0.12	-0.05	-0.08	0.42	0.15	0.44	-0.06	0.20	0.01	0.05
	FAT%	-0.32	-0.35	0.09	-0.41	-0.32	-0.26	0.25	0.11	0.68	0.46	-0.03	-0.02
	PROT%	0.01	0.09	0.19	0.09	0.02	-0.23	0.29	0.15	0.15	0.66	-0.03	0.00
	CM	0.25	0.18	0.33	0.08	0.18	0.75	-0.1	-0.01	-0.17	0.18	0.03	0.20
	SCS	0.54	0.75	-0.08	0.55	0.7	0.03	-0.11	-0.05	-0.26	0.01	0.04	0.39
Normande	FUA	0.26	0.42	0.39	0.63	0.15	0.28	0.27	0.26	-0.01	-0.03	-0.07	-0.04
	UDD	0.21	0.33	0.27	0.51	0.64	-0.53	-0.54	-0.56	-0.07	-0.23	0.00	-0.01
	FTP	-0.04	0.03	0.39	0.38	0.49	-0.13	-0.10	-0.12	0.06	0.02	-0.02	-0.02
	UB	0.16	0.11	-0.19	0.30	0.35	0.01	0.10	0.06	0.22	0.23	-0.01	-0.02
	UC	0.2	0.21	-0.08	0.72	0.33	0.03	0.00	0.02	-0.04	-0.31	0.00	-0.01
	MY	-0.53	0.16	0.06	0.1	0.06	0.61	0.90	0.95	-0.16	0.04	0.00	-0.10
	FAT	0.08	0.12	0.09	0.18	0.15	0.78	0.60	0.93	0.28	0.25	0.00	0.00
	PROT	0.01	0.37	-0.1	0.4	0.38	0.27	0.37	0.62	-0.01	0.33	0.02	-0.05
	FAT%	0.28	0.29	-0.21	0.38	0.44	0.21	0.31	0.43	0.63	0.50	-0.03	0.06
	PROT%	0.19	0.19	0.02	-0.09	0.01	0.12	0.12	0.09	0.16	0.61	-0.03	0.03
	CM	0.07	0.37	0.09	-0.01	0.05	0.01	-0.01	-0.06	0.04	0.30	0.03	0.37
	SCS	-0.01	0.06	0.00	-0.27	-0.25	0.00	-0.1	-0.18	-0.11	0.33	0.10	0.40
Holstein	FUA	0.33	0.49	-0.33	0.39	0.09	0.00	-0.02	-0.01	-0.04	-0.05	-0.07	-0.02
	UDD	0.21	0.46	-0.16	0.42	0.19	-0.07	-0.09	-0.09	-0.02	-0.06	-0.01	0.01
	FTP	-0.04	0.03	0.48	-0.29	-0.35	-0.34	-0.33	-0.34	0.06	-0.05	0.49	-0.04
	UB	0.16	0.11	-0.19	0.23	0.27	0.05	0.05	0.07	-0.01	0.09	0.12	-0.02
	UC	0.2	0.21	-0.08	0.72	0.33	-0.02	-0.01	0.00	-0.05	0.02	-0.05	0.01
	MY	0.09	0.16	0.06	0.1	0.06	0.54	0.85	0.96	-0.43	-0.03	-0.01	0.13
	FAT	0.08	0.12	0.09	0.18	0.15	0.78	0.49	0.90	0.10	0.26	0.00	-0.03
	PROT	0.01	0.37	-0.1	0.4	0.38	0.27	0.37	0.48	-0.27	0.25	-0.03	0.07
	FAT%	0.28	0.29	-0.21	0.38	0.44	0.21	0.31	0.43	0.65	0.51	0.01	-0.10
	PROT%	0.19	0.19	0.02	-0.09	0.01	0.12	0.12	0.09	0.16	0.58	-0.01	-0.10
	CM	0.07	0.37	0.59	-0.01	0.05	0.01	-0.01	-0.06	0.12	-0.03	0.02	-0.07
	SCS	-0.01	0.06	0.00	-0.27	-0.25	0.00	-0.1	-0.18	-0.04	0.04	0.33	0.38

^1^Genetic correlation = correlations based on phenotypic data as estimated using a pedigree based REML;

^2^SNP correlation = correlations estimated from AWM matrix;

^3^Trait = A yield deviation: phenotype corrected for environmental variances: FUA: fore udder attachment, UDD: udder depth development, FTP: front teat placement, UB: udder balance, UC: udder cleft, MY: milk yield, FAT and FAT%: fat yield and content, PROT and PROT%: protein yield and content, CM: clinical mastitis, SCS: Somatic cell score

Mammary morphology genetic correlations to production traits ranged from positive for fore udder attachment (FUA) and milk yield (MY) in Montbeliarde (0.42) and Normande (0.28) to medium negative for udder depth or development (UDD) and protein yield (PROT) in Normande (-0.56) and front teat placement (FTP) and fat yield (FAT) in Holstein (-0.33). SNP correlations were numerically different compared to genetic correlations; however, the correlation was generally in the same direction. For instance, the genetic correlation between fore udder attachment (FUA) and udder balance (UB) was 0.40, 0.63 and 0.39, whereas, SNP correlations for these two traits was 0.59, 0.16 and 0.16 for Montbeliarde, Normande and Holstein breeds, respectively. There were moderate to zero genetic correlations between milk yield (MY) and FUA for Montbeliarde (0.42), Normande (0.28), and Holstein (0), and low SNP correlation (Montbeliarde (0.10), Normande (0.16) and Holstein (0.16)) between MY and udder depth or development (UDD). Holstein breed had a highly positive genetic (0.49) and SNP correlation (0.59) between front teat placement (FTP) and clinical mastitis (CM), a trend that was not evident in other two breeds. However, Montbeliarde breed showed a strong SNP correlation between FTP and CM (0.33) and between UDD and CM (0.18). Other trends evident from genetic correlations were between FUA and PROT for Montbeliarde (0.39) and Normande (0.26), FUA and CM for Montbeliarde (0.19) and a moderate genetic correlation between FTP and CM for Holstein (0.49). Genetic and SNP correlations were also comparable between FTP and PROT for all breeds, with genetic/SNP correlation being from medium in Montbeliarde (0.21 / 0.12) and Holstein (-0.34 / -0.1) to low in Normande (-0.12 / -0.1). However, the trend deviated between udder balance (UB) and fat percent (FAT %) with minimal genetic correlation in Normande (0.22) and no genetic correlation in Montbeliarde and Holstein but with moderate SNP correlations in Montbeliarde (-0.41), Normande (0.38) and Holstein (0.38). In general, genetic correlations are in the range of usual values, with high correlations between milk, fat and protein, a moderately negative correlation between production and type traits, moderately positive correlations between conformation traits, and low correlations otherwise. However, though there were deviations between genetic and SNP correlations in some of the traits, most traits correlations were in the same direction thus drawing plausibility of SNP correlated traits.

### Gene ontology (GO) for AWM selected genes

**Fig 4:**
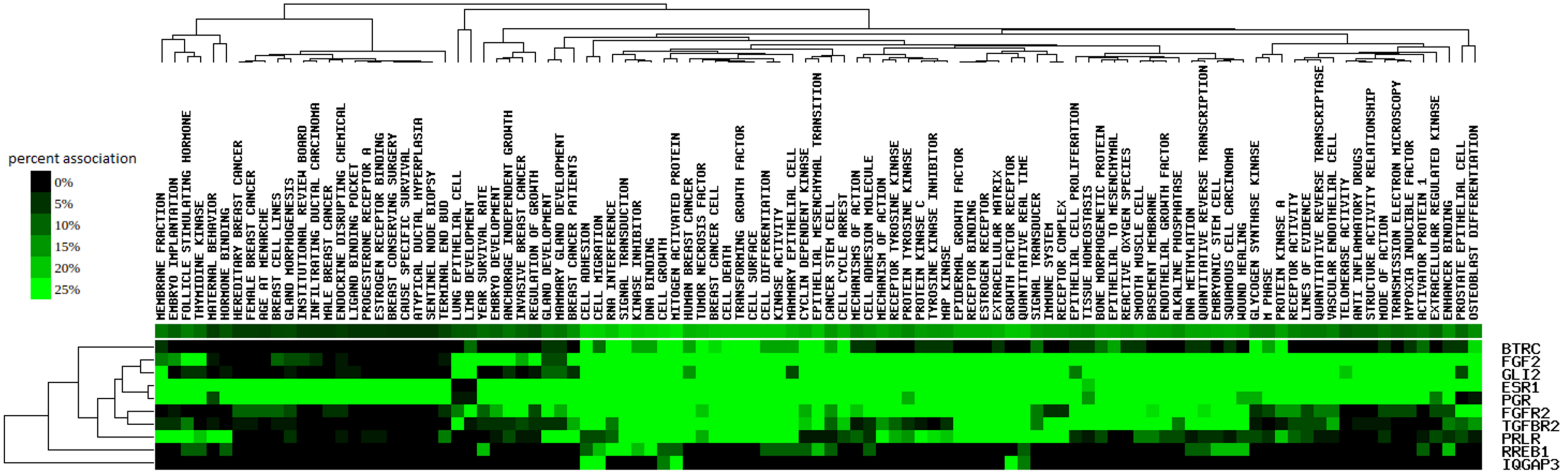
Ten candidate gene-GO term interactions with percentage association in three French dairy breeds.

We considered main ontology domains including, biological processes, cellular component, and molecular functions. We observed 39 gene clusters (S2 Table) and Table 5 presents the top five biological processes that are relevant for udder morphology and health traits (S3 Table contains complete gene ontology list for this study). Top cluster had eight GO terms with the most significant GO term being “mammary gland epithelium development” (p = 4.64x10^-16^). Among the AWM-PCIT, genes ten were transcription factors (TF) directly associated with the terms “mammary gland development”, “mammary gland duct morphogenesis", mammary gland alveolus development”, “tissue development”, and, “epithelial tube morphogenesis”. These ten TF include *GLI2* (BTA2:72.98Mb), *IQGAP3* (BTA3:14.31Mb), *PGR* (BTA4:62.53Mb), *ESR1* (BTA9:89.97Mb), *FGF2* (BTA17:35.23Mb), *PRLR* (BTA20:39.13Mb), *TGFBR2* (BTA22:5.14Mb), *RREB1* (BTA23:47.90Mb), *BTRC* (BTA26:22.06Mb), and, *FGFR2* (BTA26:41.82Mb). Fig 4 presents their GO term interactions with percentage association. Other GO terms in the top cluster included “gland development” (p = 6.50x10^-12^) and “system development” (p = 5.38x10^-5^). Top GO term for the second cluster was “mammary gland epithelium development” (p = 5.16x10^-16^) while “regulation of intracellular signal transduction” was the least significant term in this cluster (p = 4x10^-4^). There was an enrichment for “neuropathic pain-signaling in dorsal horn neurons pathway” (p = 2.57x10^-8^), “G-protein coupled receptor signaling” (p = 1.07x10^-7^) as well as the “CREB signaling in neurons” (p = 7.24x10^-7^). Other pathways detected were “cAMP-mediated signaling” (p = 2.88x10^-5^), “synaptic long-term depression (p = 8.71x10^-7^), “axonal guidance signaling” (p = 1.15x10^-6^), and “synaptic long-term potentiation” (p = 1.58 x10^-5^).

**Table 5:** Top five clusters of transcription factors/genes and associated GO terms associated with udder morphology in three French dairy breeds.

Transcription factor^1^/genes GO^2^ Term	Gene count	p	Top Associated Genes^3^
cluster1 Enrichment Score^4^: 9.17			
mammary gland epithelium development	134	4.64 x10^-16^	[BTRC, ESR1, FGF2, FGFR2, GLI2, IQGAP3, PGR, PRLR, RREB1, TGFBR2]
regulation of response to stimulus	3653	7.14 x10^-6^	[BMP7, GCH1]
gland development	429	6.50 x10−^12^	[ROR2, RORA, SYCP2, TCF7L2]
epithelium development	1057	6.64 x10^-9^	[FGFR2, HHIP, RDH10, RSPO2, TNC]
tissue development	1714	2.10 x10^-7^	[AKT3, CTNNA2, CTNNA3, GNAI3, INADL, MAGI3, PARD3, PPP2R2B, PRKCB, PRKCE, PRKCH, PTEN, RAB3B]
animal organ development	3022	2.66 x10^-7^	[ADGRB1, ADGRB3, ANGPT1, BMPER, CALCRL, COL8A1, CSPG4, EPAS1, FGF2, FGFR2, FN1, GJA5, HDAC9, HSPG2, LOXL2, MAP2K5, MTDH, NOV, NRP2, NRXN3, PDCL3, PRKCB, PTEN, PTK2, ROCK2, RORA, SH2D2A, SHB, SPI1, STAB1, TGFBR2, THSD7A, TIE1, VAV3]
system development	4309	5.38 x10^-5^	[HMGCLL1, HMGCS2, OXCT1]
multicellular organism development	4855	2.00 x10^-4^	[GALNT13, GALNT14, GALNT18, GALNTL6, GCNT3]
cluster2 Enrichment Score^4^: 8.92			
mammary gland alveolus development	68	5.16 x10^-16^	[ESR1, PRLR]
intracellular signal transduction	2681	6.97 x10^-5^	[ABCA1, SYCP2]
regulation of intracellular signal transduction	1632	4 x10^-4^	[ANXA4, CD58]
cluster3 Enrichment Score^4^: 8.41			
positive regulation of macromolecule metabolic process	2863	2.77 x10^-9^	[CHRNB2, GPAM, IL7, PELI1, SPTA1, STAT5B, VAV3, VTCN1]
positive regulation of cellular metabolic process	2899	2.62 x10^-9^	[ARPC2, AXIN2, BAIAP2, CDH4, CUX1, CUX2, EPB41L5, EPHA4, FBXW8, FN1, FYCO1, LPAR3, LRP8, LRRC16A, MAP2K2, NTRK2, PTPRD, RREB1, RUFY3, SEMA4D, TCF7L2, TGFB3, TGFBR2]
positive regulation of metabolic process	3563	1.46 x10^-7^	[PIWIL2, PLCB1]
negative regulation of biological process	4579	8.50 x10^-6^	[DAB1, FYCO1, PTEN, PTK2, RTN4, RUFY3, SEMA3B, SEMA4D, SYNGAP1]
cluster4 Enrichment Score^4^: 8.26			
branching morphogenesis of an epithelial tube	146	5.32 x10^-9^	[CD44, EYA1, FAT4, FGF2]
morphogenesis of a branching epithelium	178	1.29 x10^-8^	[ADORA1, ARRB1, CACNA1A, CACNA1B, GABBR2, GABRA1, GNAI3, GNB5, GNG7, GNGT2, PDE11A, PDE1A, PDE2A, PRKACB, PRKCB]
epithelial tube morphogenesis	305	1.65 x10^-7^	[FGFR2, HHIP, RDH10, RSPO2, TNC]
tube morphogenesis and development	341	2.28 x10^-7^	[AARS2, ALKBH8, CDK5RAP1, CDKAL1, NDC1, NUP210, NUP93, SARS, TSEN54]
organ morphogenesis	905	2.63 x10^-5^	[CHRNB2, KCNA2]
cluster5 Enrichment Score^4^: 7.99			
cellular response to organic substance	2352	2.89 x10^-9^	[LBP, NDUFA2]
cellular response to chemical stimulus	2845	1.28 x10^-7^	[ABCC9, AKAP6, CACNA1D, CASQ2, HCN1, KCNA2, KCNAB2, KCNB2, KCNC1, KCND3, KCNH1, KCNK2, KCNMA1, KCNMB1, KCNN2, KCNN3, KCNT1, KCNT2, KCNV2, STK39, YWHAE]
response to organic substance	2981	2.47 x10^-7^	[ASS1, SMYD3, SRD5A1, TRIM63]
response to chemical	4373	6.51 x10^-5^	[MCM3, POLD1, POLE3, PRIM2, SMARCAL1]

^1^Transcription factor = a protein that controls rate of transcription of genetic information from DNA to messenger RNA, by binding to a specific DNA sequence. Sometimes its homonymous to gene;

^2^GO = Gene Ontology as expressed in main domains: Biological, Cellular and Molecular;

^3^Top Associated Genes = most probable associated candidate gene of interest for udder morphology;

^4^Enrichment Score = measure of over-represented (or under-represented) GO terms using AWM gene annotations

Pathway enrichments detected (S3 File) included “Calcium: cation antiporter activity” and the “Calcium-activated Potassium channel activity” for molecular functions (p<10^−3^), whereas, for biological processes, “dendrite development” and “putrescine biosynthetic” process were most represented (P<10^−3^) while “postsynaptic density” and “presynaptic membrane” were top GO terms for cellular components.

## Discussion

Previously, Fortes et al. [8] and Ramayo-Caldas et al. [23] suggested the Association Weight Matrix’s (AWM) as an alternative tool to identify genes that would otherwise be missed by traditional single-trait GWAS. This study further supports that suggestion by focusing on GWAS for other kinds of traits, such as udder morphology and health traits common across three French dairy breeds. Though single-trait-single-SNP GWAS focus is on most significant SNP, they can, aid in identifying lead SNP for QTL associated with a given trait. Our study identified three lead SNP associated with front teat placement (FTP) and fore udder attachment (FUA). These SNP mapped *FGF2*, *PRLR*, and *BTRC* genes. *PRLR* gene (prolactin receptor) was previously associated with milk production traits in Finnish Ayrshire dairy cows [24]. Wang et al. [25] reported the association of *FGF2* gene (Fibroblast growth factor 2) to fat yield and percentage and somatic cell score in US Holstein. Coleman-Krnacik et al. [26] reported the expression of *FGF2 gene* in the bovine mammary gland and uterine endometrium (UE). In the mammary gland, the *FGF2* gene may play a role in development and reorganization of the mammary gland, while in UE, *FGF2* gene is mainly expressed throughout estrous cycle and early pregnancy. *BTRC* gene (Beta-Transducing Repeat Containing E3 Ubiquitin Protein Ligase) is an F-box protein involved in Wnt/β-Catenin signaling pathway and indirectly activates nuclear factor kappa-B (NF-kB)[27]. Raven et al. reported these pathways to be highly relevant during mammary development and pregnancy, and as such, could have a major functional role in lactation [27]. The AWM-PCIT algorithm identified interacting candidate genes for udder conformation traits by first establishing SNP based correlation and fixing udder depth or development (UDD) as the key trait when constructing the AWM SNP matrix. These genes were represented by several biological processes involved in positive regulation of cellular biosynthetic processes and cell development, suggesting an endogenous characterization linked to udder morphology [28]. Fortes et al. [10] reported more similar SNP and genetic correlations for traits with moderate to high heritability and less similar correlations between traits with low heritability (Table 4). In our study, most traits had a heritability >10% thus aiding both GWAS detection power and AWM SNP detection.

The AWM was assessed for gene ontology (GO). The top GO terms were Calcium cation antiporter and Calcium-activated Potassium channel activity. As reported by Paulsen et al. [29], the former is a member of the cation diffusion facilitator (CDF) superfamily which are integral membrane proteins that increase tolerance to divalent metal ions, whereas, the latter is involved in ionic signaling in cells, a critical function for hormonal control of cell proliferation and differentiation [30]. Control of calcium signaling is likely to have profound effects on mammary physiology and pathophysiology. Their high significance level can be explained by the fact that mammary glands extract large quantities of calcium from the plasma during lactation [31], to ensure sufficient calcium concentration in milk. Dendrite development and putrescine biosynthetic processes were top biological GO terms. Dendritic cells (DC) are accessory cells of the mammalian immune system whose work is availing antigen material to T cells of the immune system [32–33]. This ability to stimulate native T-cells makes this result significant because it can directly be applied to improve udder health. DC has also been reported to play a pivotal role in the initiation of an adaptive immune response [34]. Putrescine, as a member of polyamine pathway, is regulated by the periovulatory endocrine milieu [35].

The central pathways that showed enrichment were “Neuropathic pain-signaling in dorsal horn neurons pathway," and, “CREB signaling pathway." Neuropathic pain is the pain after nerve injury whereas the dorsal horn (tip of the spinal cord) pathways have been shown to offer substantial overlap with spinal projections from adjacent mammary glands in model organisms [36]. Reflex milk ejection may result from the strong integration of sensory input from mammary glands afferents that terminate in the dorsal horn. CREB (cyclic-AMP response element-binding) protein family of transcription factors (TF—a protein that controls the rate of transcription of genetic information from DNA to messenger RNA, by binding to a specific DNA sequence [37]) play a crucial role in supporting the survival of sensory neurons [38]. The intervention of sensory neurons stimulates cells to secrete nerve growth factor (NGF) via the sympathetic nervous system (SNS) that maintains homeostasis [39]. NGF mediate its functions through ligation of tyrosine kinase (Trk) receptors [40]. Regulation of Trk signaling is by a variety of intracellular signaling cascades, which include MAPK pathway, which promotes cell continuation and growth [41]. Previous studies indicate that Trk could be multifunctional growth factors that exert various effects through their receptors on non-neuronal cells such as mammary ducts [30].

Fontanesi et al. [8] reported that long-term transcriptomic adaptations of tissues depend on the action of external stimuli that induce action of cellular functions on transcription factors (TF) [42]. In this study, we identified 39 interacting gene clusters and the most significant cluster had ten TF directly involved with mammary gland development and mammary gland duct morphogenesis: *BTRC*, *ESR1*, *FGF2*, *FGFR2*, *GLI2*, *IQGAP3*, *PGR*, *PRLR*, *RREB1*, and, *TGFBR2*. These TF are homonymous with genes encoding them. This top cluster is directly involved in mammary gland development, regulation of response to a stimulus, gland development, epithelium development, tissue development, animal organ development, system development and multicellular organism development. This was partly in agreement to works reported by Yang et al. [43] on QTL associated with follicle stimulating hormone production in Chinese Holstein cattle.

## Conclusion

Our study suggests the usefulness of system-based approaches to identify candidate genes from interacting gene networks in a multi-breed context. We achieved this by exploiting associations between correlated traits. The reluctant inclusion of intergenic SNP leaves the possibility that the AWM approach was not capturing significant SNP such as trans-activators. Nonetheless, the AWM proved to be more efficient for integrating related complex traits and analyzing thousands of SNP and therefore appropriate for the analysis of these complex traits. When applied to our dataset, it predicted gene interactions that are consistent with the known biology of udder morphology and health captured known TF (e.g., *ESR1*, *FGF2*, *GLI2*, *PGR* and *BTRC*), and provided new candidate genes for udder morphology. This experiment may be replicated using whole genome sequence data and other independent datasets.

## Supporting information

S1 Fig Manhattan plots for milk production, udder morphology and udder health traits in three French dairy cattle breeds. (PDF)

S1 Table Associated Weight Matrix for three French dairy cattle breeds. (XZ)

S2 Table Enrichment scores for udder morphology genes. (XLSX)

S3 Table Gene ontology for AWM-PCIT selected genes. (XLSX)
