# Supplementary material for: A system-based analysis of the genetic determinism of udder conformation and health phenotypes across three French dairy cattle breeds
Source: PLoS One. 2018 Jul 2;13(7):e0199931. doi: 10.1371/journal.pone.0199931 (PMC6028091; doi:10.1371/journal.pone.0199931)
Supplement: S1 Fig — (PDF) [file pone.0199931.s001.pdf]

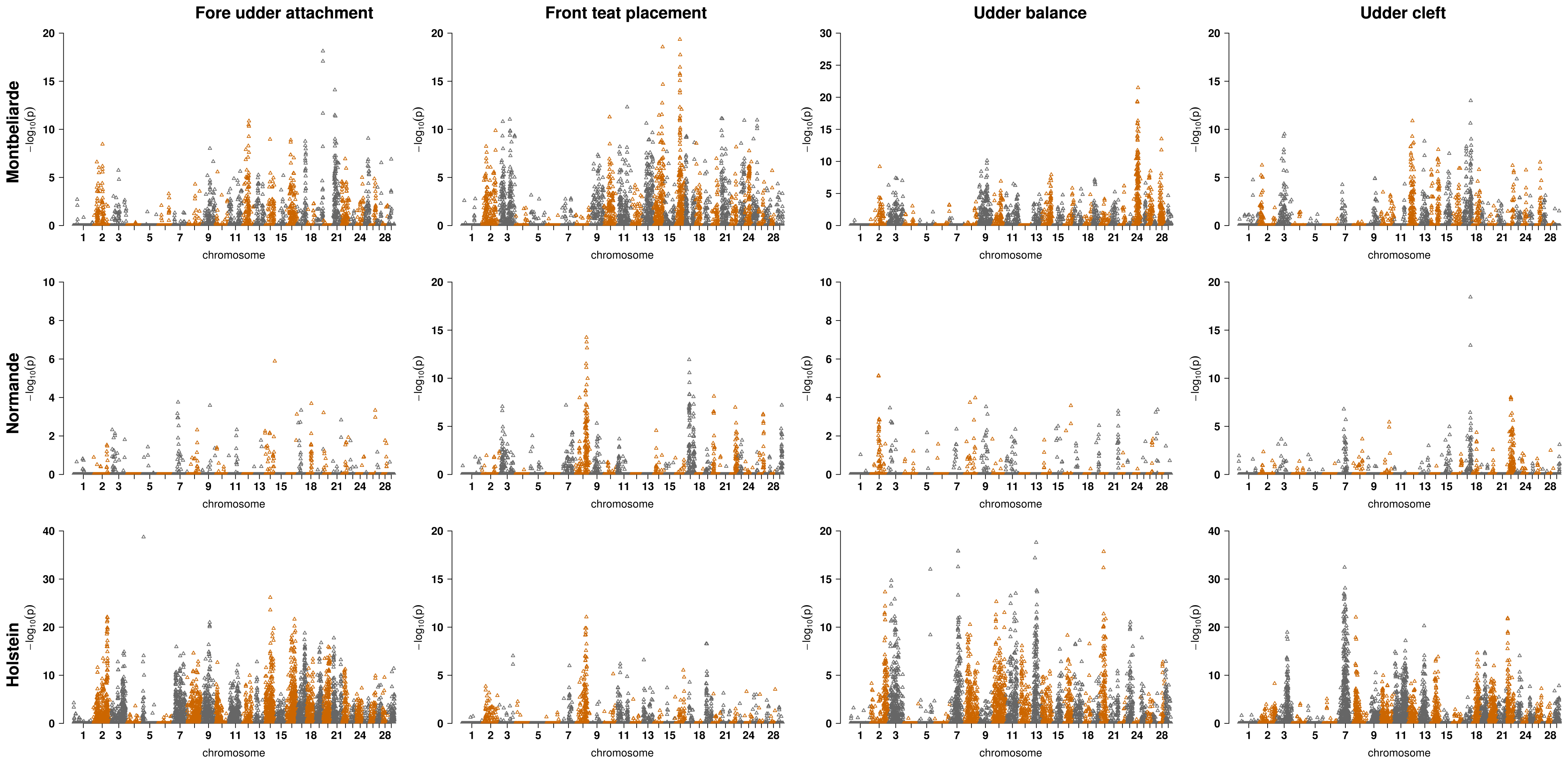

Manhattan plots for udder traits in three French dairy breeds

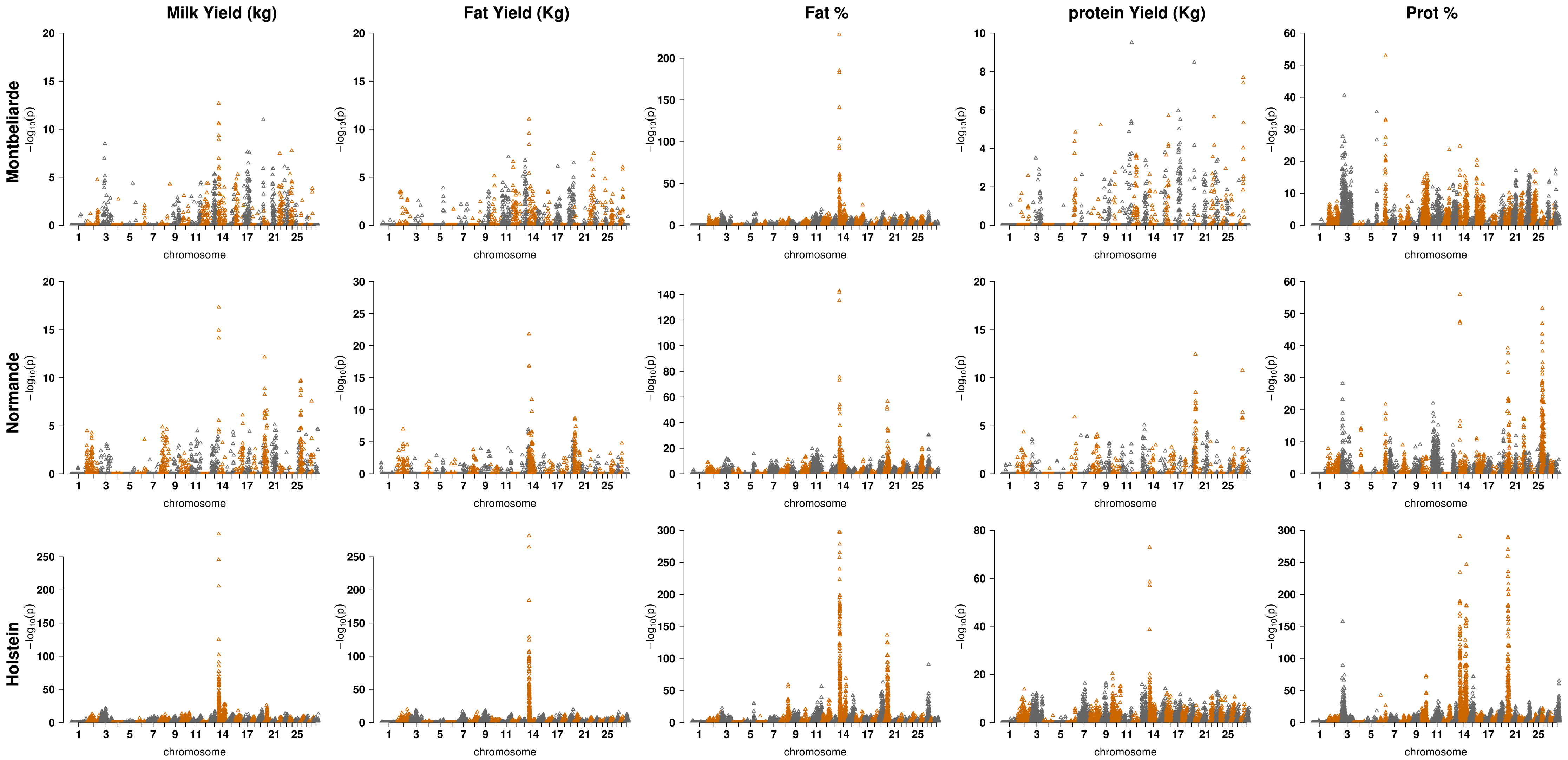

Manhattan plots for five milk production traits in three French dairy breeds

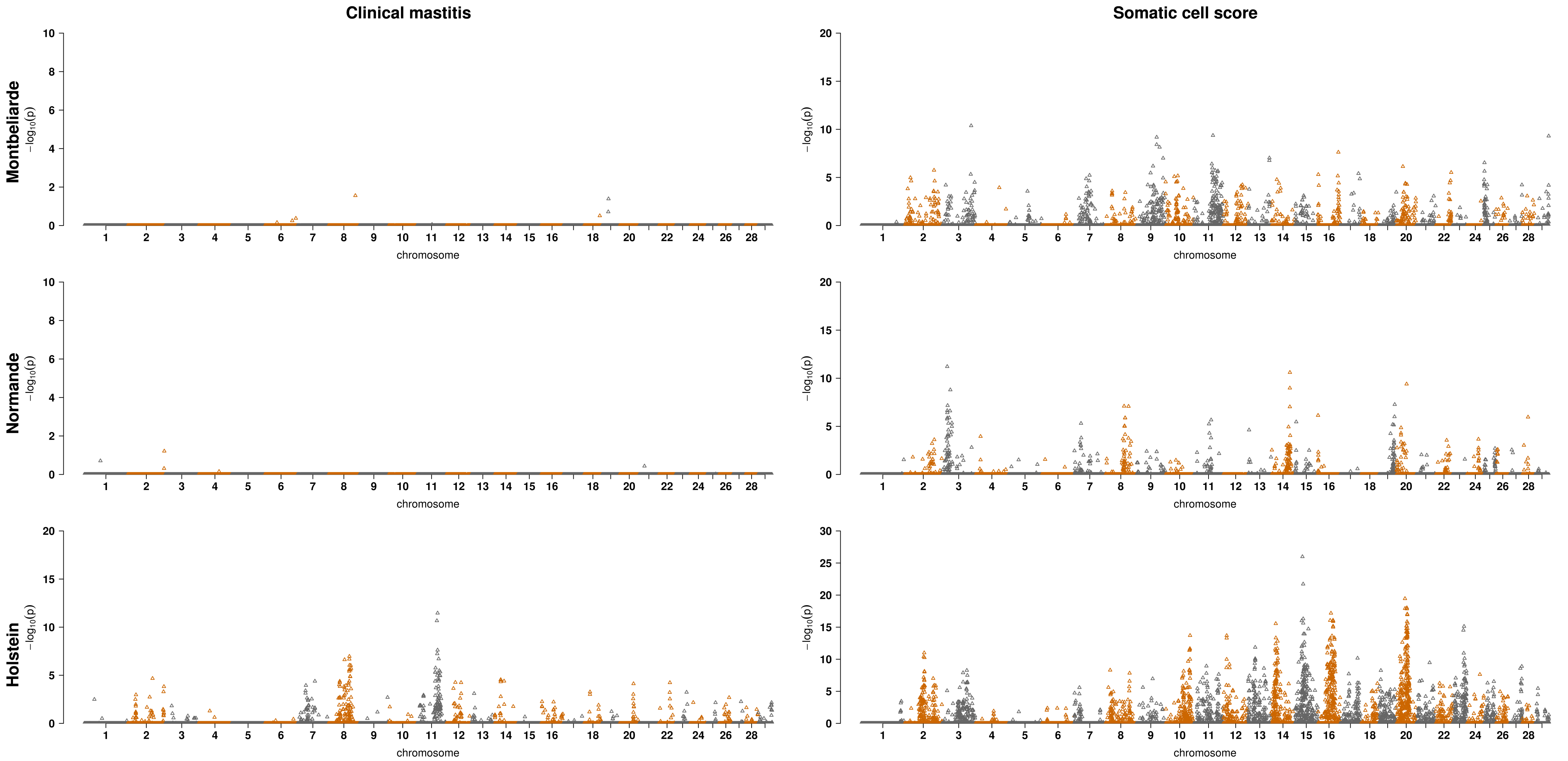

Manhattan plots for udder health traits in three French dairy breeds
